# Supplementary material for: Formulation and Biological Evaluation of Mesoporous Silica Nanoparticles Loaded with Combinations of Sortase A Inhibitors and Antimicrobial Peptides
Source: Pharmaceutics. 2022 May 4;14(5):986. doi: 10.3390/pharmaceutics14050986 (PMC9144937; doi:10.3390/pharmaceutics14050986)
Supplement: Supplementary file 1 [file pharmaceutics-14-00986-s001.zip › pharmaceutics-1694152-supplementary.pdf]

Supplementary Materials: Formulation and Biological Evaluation of Mesoporous Silica Nanoparticles Loaded with Combinations of Sortase A Inhibitors and Antimicrobial Peptides

Sitah Alharthi, Zyta M. Ziora, Taskeen Janjua, Amirali Popat, Peter M. Moyle

Table S1. The concentrations of the sortase A inhibitor (SrtAIs; TC, CUR, QC, and BR)-loaded MCM-41 and MCM-41-PO_3^- MSNs, AMPs (PEX, INDO, and MASTO), and their combinations used in the cell viability study. Red values are the MICs of each compound.

Compound	Concentrations (µg/mL)					
PEX	50.0	25.0	12.5	6.25	3.13	1.56
INDO	150	75.0	37.5	18.8	9.38	4.69
MASTO	25.0	12.5	6.26	3.13	1.57	0.78
TC MCM-41	1000	500	250	125	62.5	31.3
CUR MCM-41	500	250	125	62.5	31.3	15.6
QC MCM-41	500	250	125	62.5	31.3	15.6
BR MCM-41	1000	500	250	125	62.5	31.3
TC MCM-41-PO_3^-	500	250	125	62.5	31.3	15.6
CUR MCM-41-PO_3^-	250	125	62.5	31.3	15.6	7.81
QC MCM-41-PO_3^-	500	250	125	31.3	31.3	15.6
BR MCM-41-PO_3^-	500	250	125	62.5	31.3	15.6
PEX + TC MCM-41	12.5 + 125	6.25 + 62.5	3.13 + 31.3	1.56 + 15.6	0.78 + 7.81	0.39 + 3.91
PEX + BR MCM-41	12.5 + 62.5	6.25 + 31.3	3.13 + 15.6	1.56 + 7.81	0.79 + 3.91	0.39 + 1.95
INDO + QC MCM-41	37.5 + 125	18.8 + 62.5	9.38 + 31.3	4.68 + 15.6	2.34 + 7.81	1.17 + 3.91
MASTO + CUR MCM-41	25.0 + 62.5	12.5 + 31.3	6.25 + 15.6	3.13 + 7.81	1.56 + 3.91	0.78 + 1.95
PEX + TC MCM-41-PO_3^-	12.5 + 62.5	6.25 + 31.3	3.13 + 15.6	1.56 + 7.81	0.79 + 3.91	0.39 + 1.95
PEX + BR MCM-41-PO_3^-	12.5 + 62.5	6.25 + 31.3	3.13 + 15.6	1.56 + 7.81	0.79 + 3.91	0.39 + 1.95
INDO + QC MCM-41-PO_3^-	37.5 + 125	18.8 + 62.5	9.38 + 31.3	4.68 + 15.6	2.34 + 7.81	1.17 + 3.91

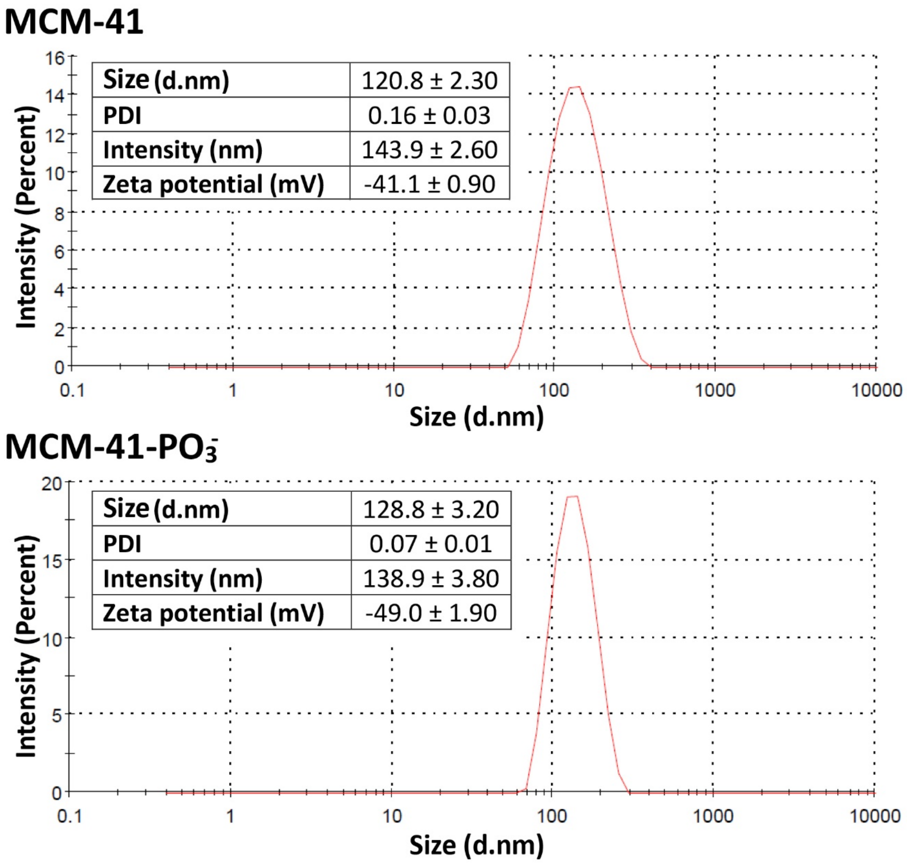

Figure S1. Size, polydispersity index (PDI), intensity mean, and zeta potential of MCM-41 and MCM-41 PO_3^- MSNs.

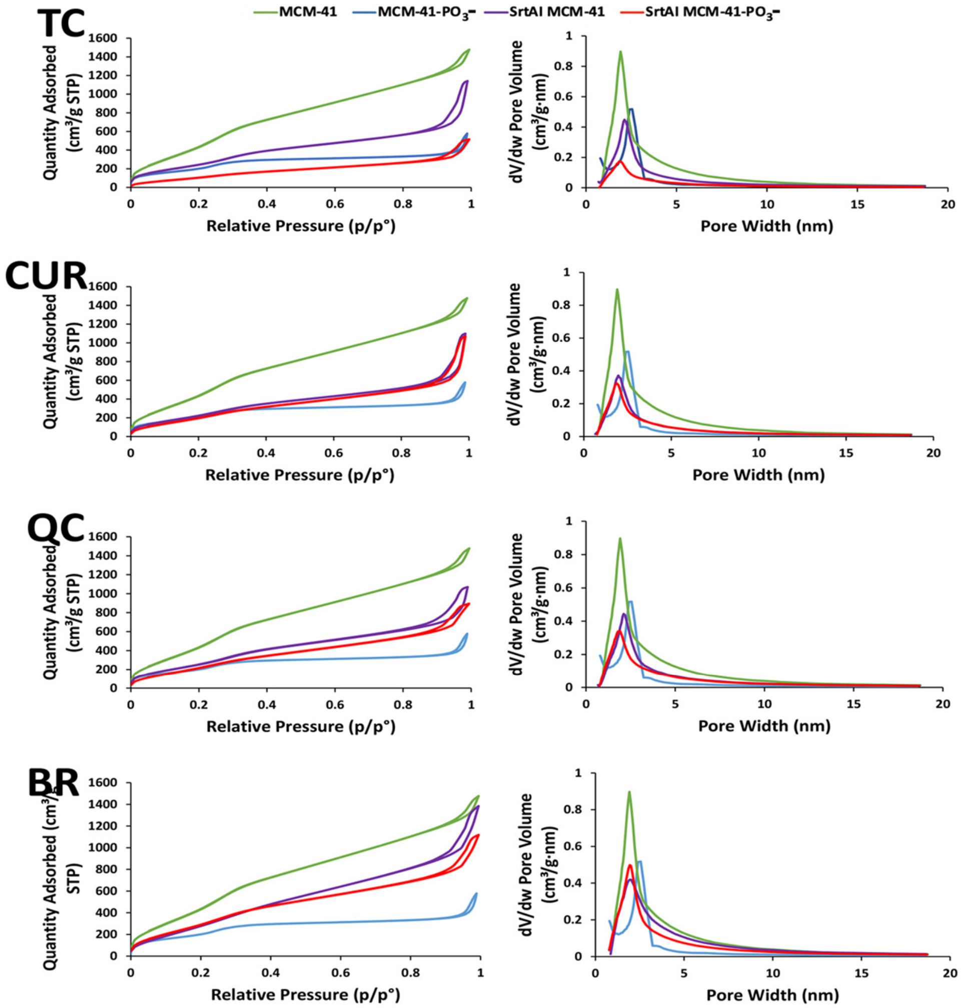

Figure S2. N_2-physisorption isothermal analysis and pore size distribution of SrtAI-loaded and non-loaded MCM-41 and MCM-41-PO_3^- MSNs.

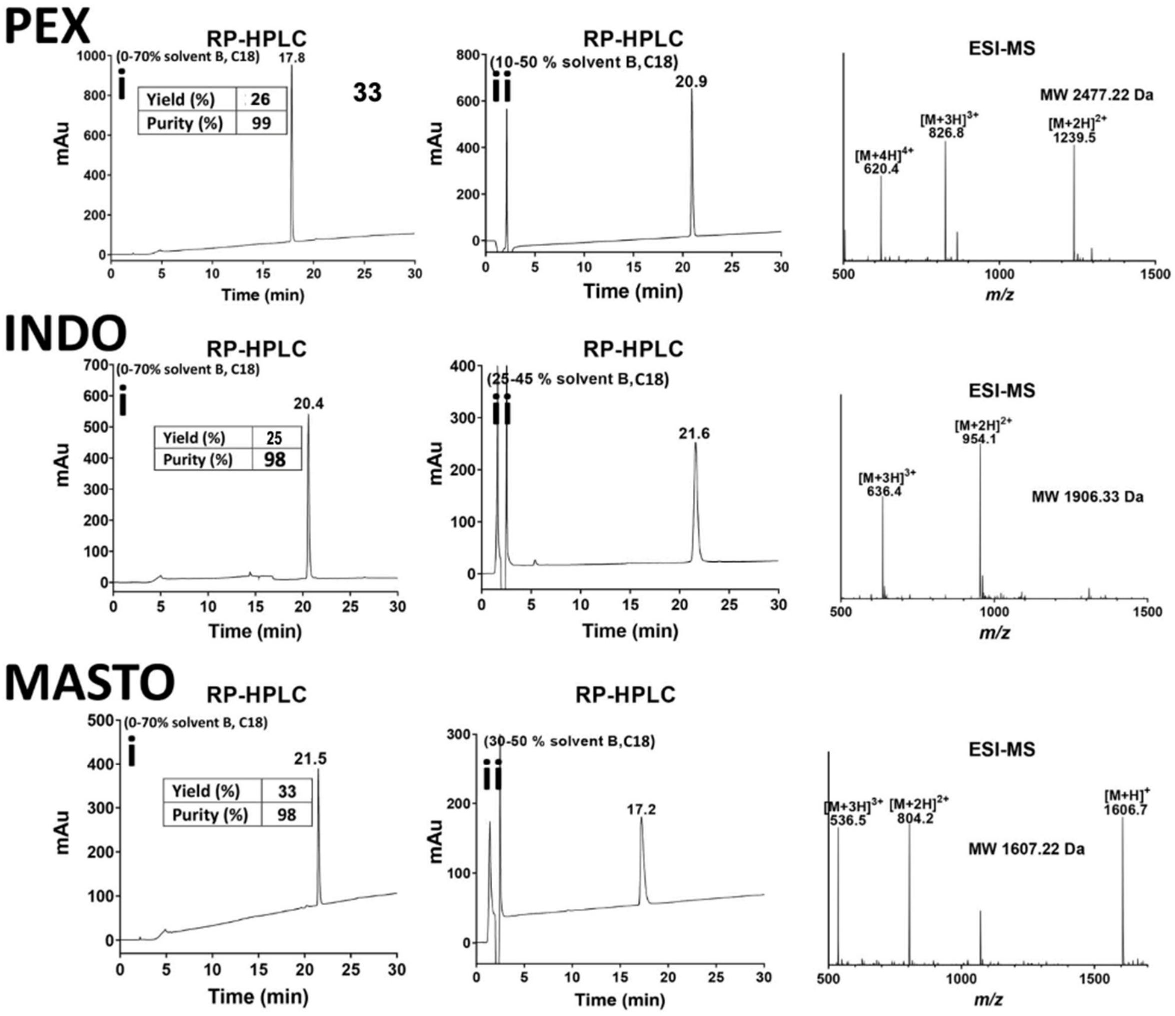

Figure S3. (**i**) Wide and (**ii**) narrow RP-HPLC gradients and ESI-MS data for purified PEX, INDO, and MASTO AMPs.

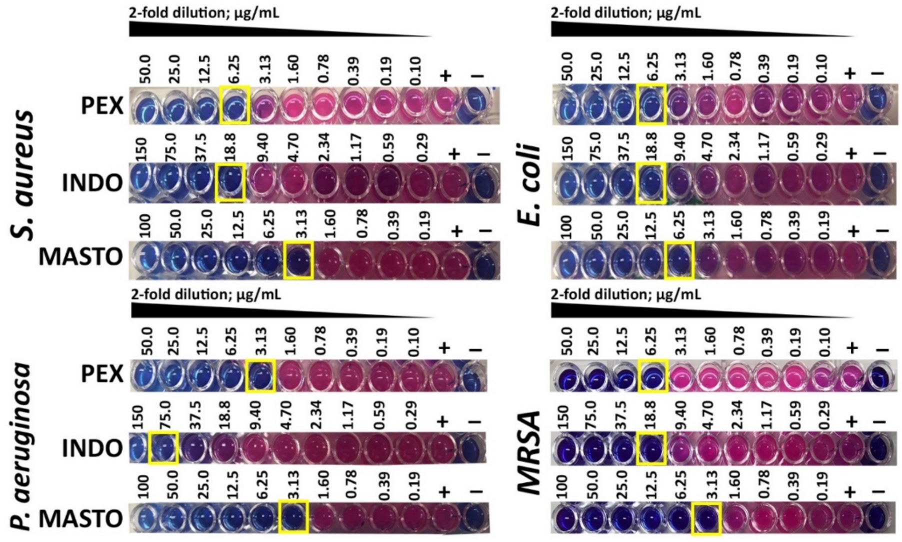

Figure S4. MICs of PEX, INDO, and MASTO against *S. aureus* ATCC 25923, *E. coli* ATCC 25922, *P. aeruginosa* ATCC27853, and MRSA ATCC43300 using the BMD assay.

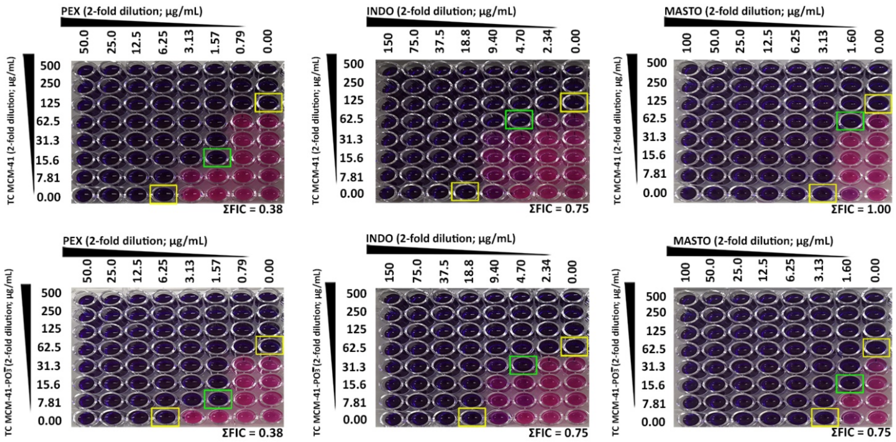

Figure S5. Fractional inhibitory concentration (FIC) index of TC MCM-41 and MCM-41-PO_3^- in combination with antimicrobial peptides (AMPs; PEX, INDO, and MASTO) *against S. aureus* ATCC25923 using checkerboard assay.

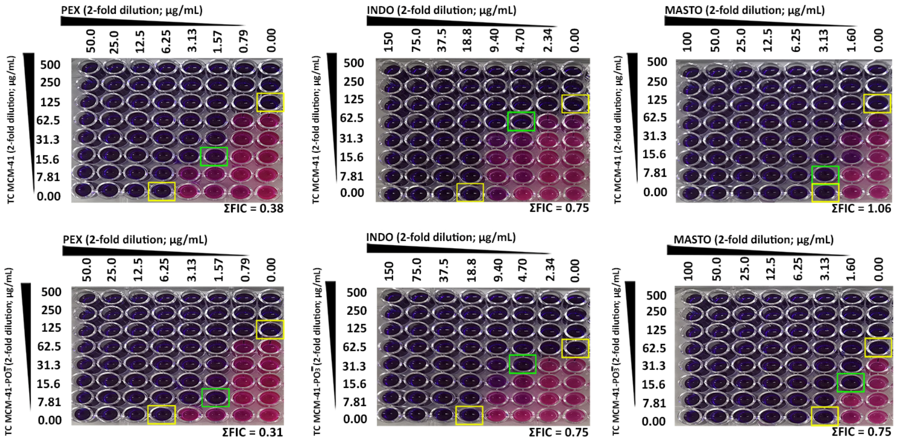

Figure S6. Fractional inhibitory concentration (FIC) index of TC MCM-41 and MCM-41-PO_3^- in combination with antimicrobial peptides (AMPs; PEX, INDO, and MASTO) against MRSA ATCC43300 using checkerboard assay.

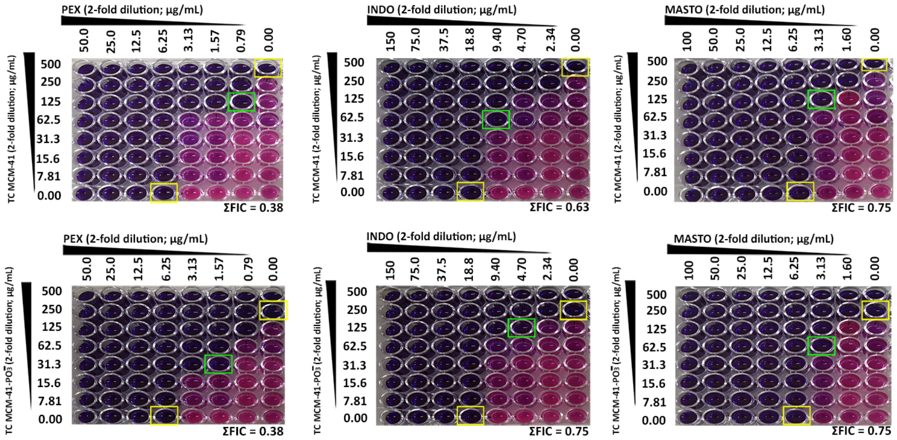

Figure S7. Fractional inhibitory concentration (FIC) index of TC MCM-41 and MCM-41-PO_3^- in combination with antimicrobial peptides (AMPs; PEX, INDO, and MASTO) against E. coli ATCC25922 using checkerboard assay.

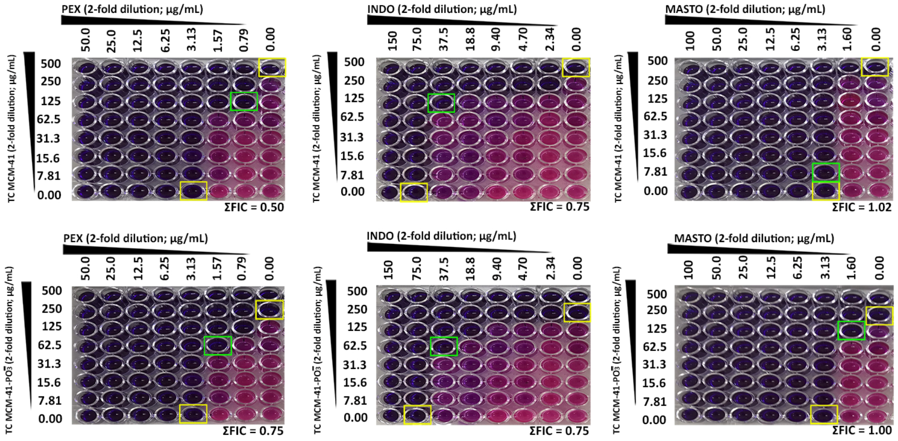

Figure S8. Fractional inhibitory concentration (FIC) index of TC MCM-41 and MCM-41-PO_3^- in combination with antimicrobial peptides (AMPs; PEX, INDO, and MASTO) against P. aeruginosa ATCC27853 using checkerboard assay.

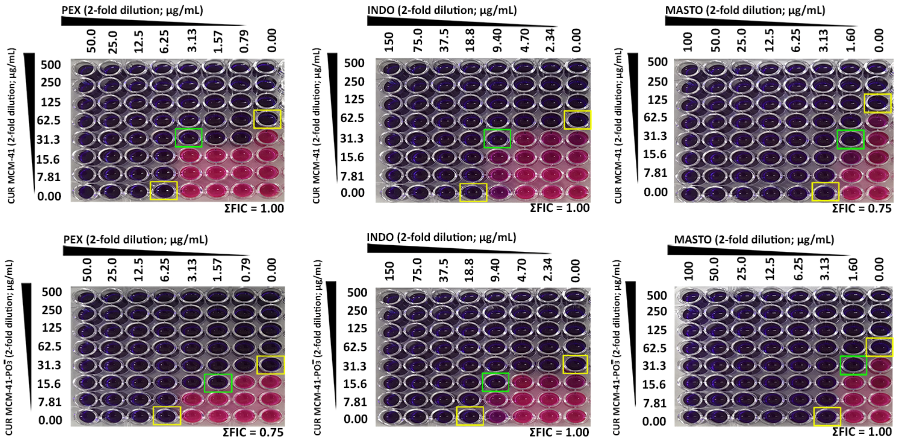

Figure S9. Fractional inhibitory concentration (FIC) index of CUR MCM-41 and MCM-41-PO_3^- in combination with antimicrobial peptides (AMPs; PEX, INDO, and MASTO) against S. aureus ATCC25923 using checkerboard assay.

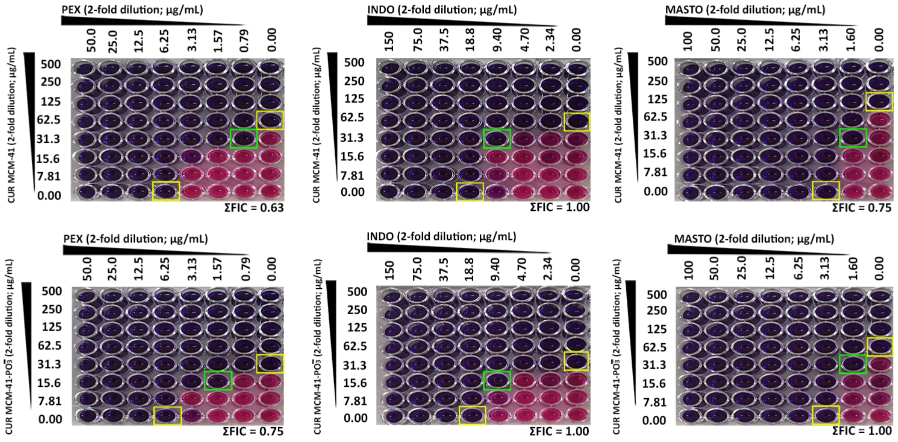

Figure S10. Fractional inhibitory concentration (FIC) index of CUR MCM-41 and MCM-41-PO_3^- in combination with antimicrobial peptides (AMPs; PEX, INDO, and MASTO) against MRSA ATCC43300 using checkerboard assay.

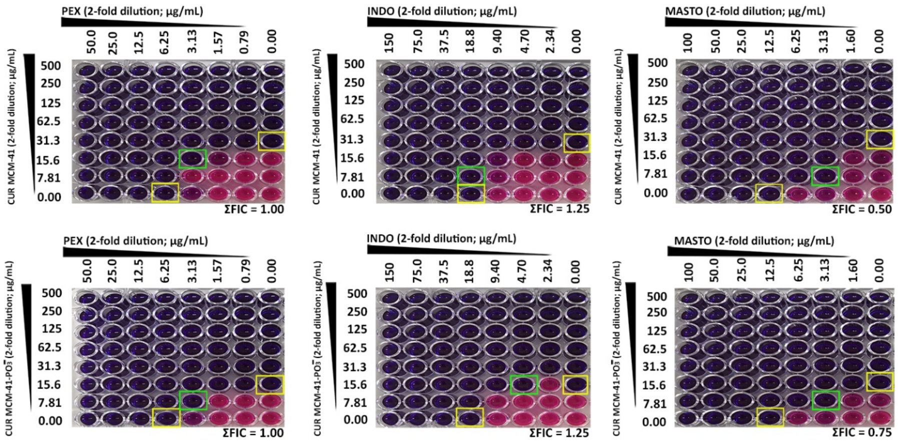

Figure S11. Fractional inhibitory concentration (FIC) index of CUR MCM-41 and MCM-41-PO_3^- in combination with antimicrobial peptides (AMPs; PEX, INDO, and MASTO) against E. coli ATCC25922 using checkerboard assay.

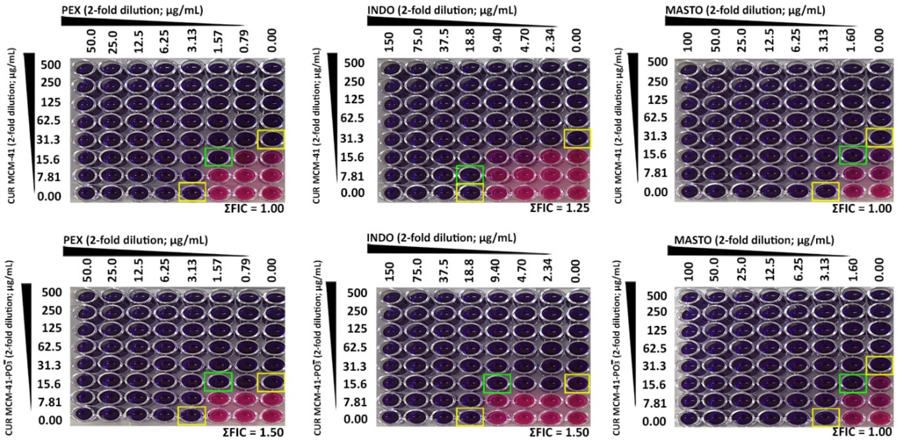

Figure S12. Fractional inhibitory concentration (FIC) index of CUR MCM-41 and MCM-41-PO_3^- in combination with antimicrobial peptides (AMPs; PEX, INDO, and MASTO) against P. aeruginosa ATCC27853 using checkerboard assay.

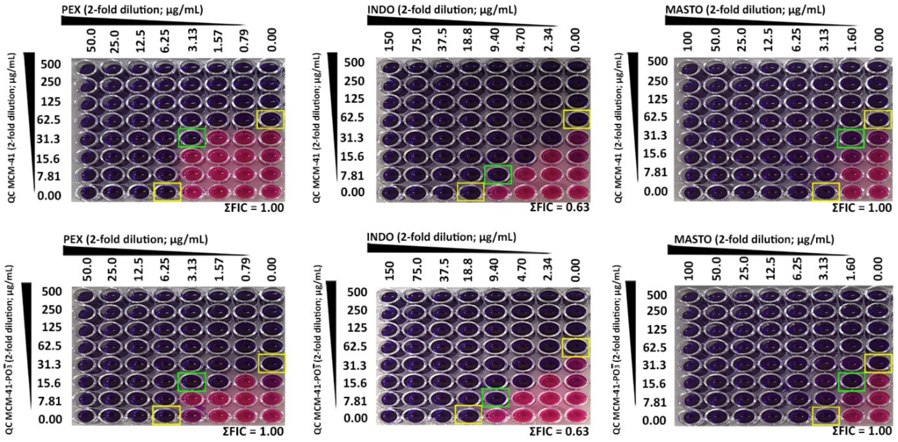

Figure S13. Fractional inhibitory concentration (FIC) index of QC MCM-41 and MCM-41-PO_3^- in combination with antimicrobial peptides (AMPs; PEX, INDO, and MASTO) against S. aureus ATCC25923 using checkerboard assay.

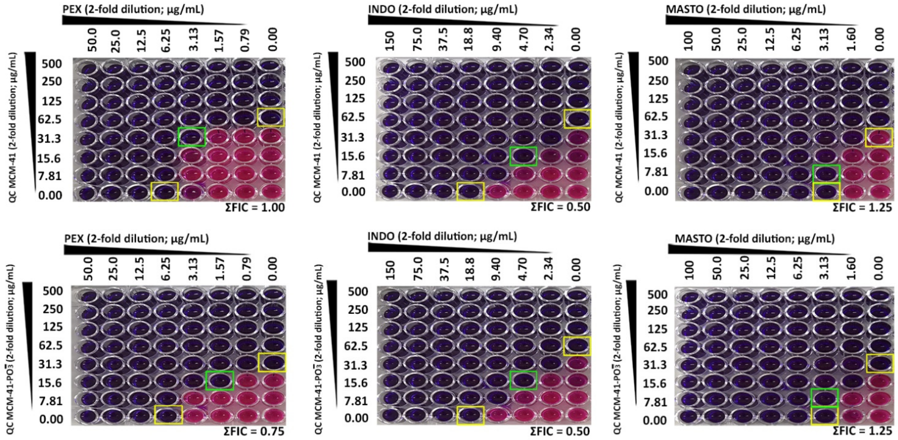

Figure S14. Fractional inhibitory concentration (FIC) index of QC MCM-41 and MCM-41-PO_3^- in combination with antimicrobial peptides (AMPs; PEX, INDO, and MASTO) against MRSA ATCC43300 using checkerboard assay.

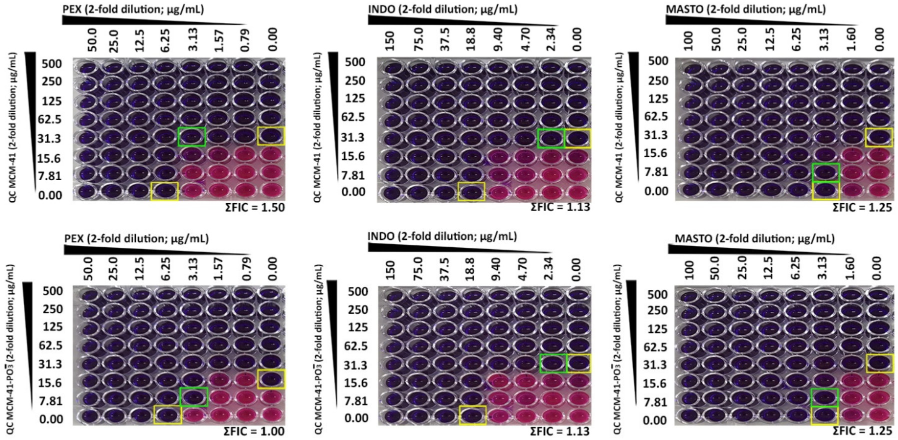

Figure S15. Fractional inhibitory concentration (FIC) index of QC MCM-41 and MCM-41-PO_3^- in combination with antimicrobial peptides (AMPs; PEX, INDO, and MASTO) against *E. coli* ATCC25922 using checkerboard assay.

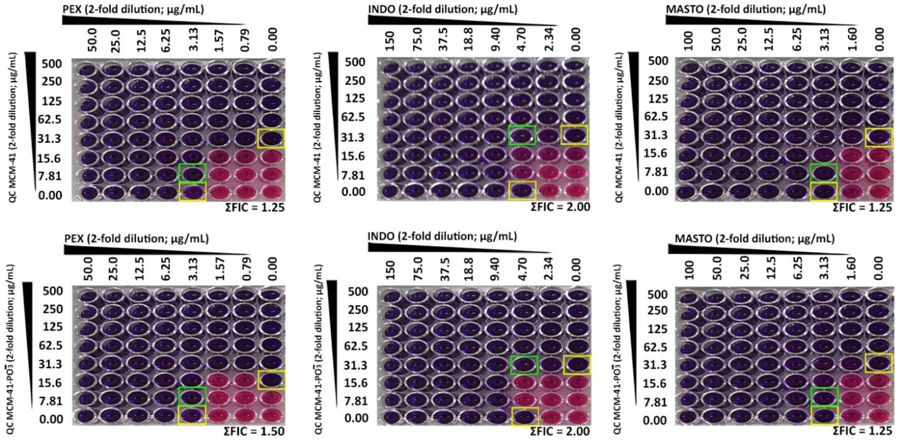

Figure S16. Fractional inhibitory concentration (FIC) index of QC MCM-41 and MCM-41-PO_3^- in combination with antimicrobial peptides (AMPs; PEX, INDO, and MASTO) against P. aeruginosa ATCC27853 using checkerboard assay.

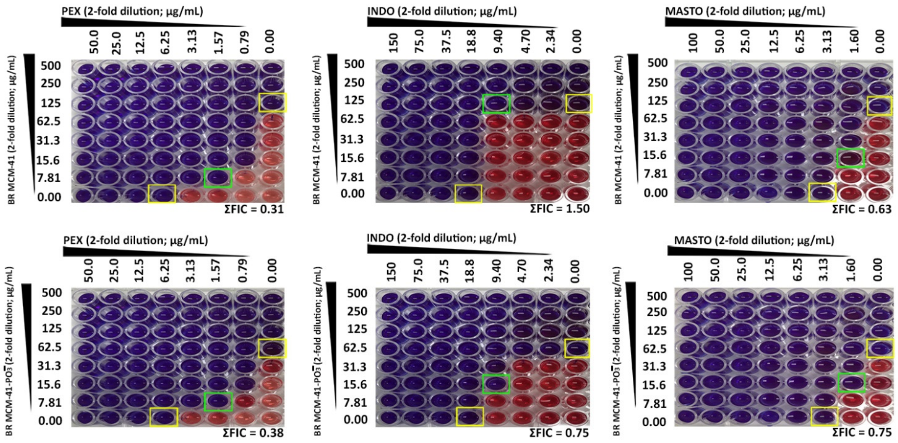

Figure S17. Fractional inhibitory concentration (FIC) index of BR MCM-41 and MCM-41-PO_3^- in combination with antimicrobial peptides (AMPs; PEX, INDO, and MASTO) against MRSA ATCC43300 using checkerboard assay.

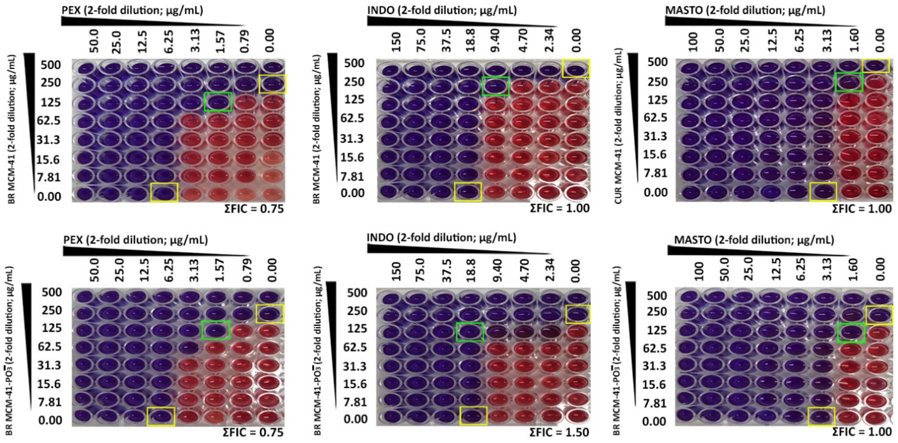

Figure S18. Fractional inhibitory concentration (FIC) index of BR MCM-41 and MCM-41-PO_3^- in combination with antimicrobial peptides (AMPs; PEX, INDO, and MASTO) against E. coli ATCC 25922 using checkerboard assay.

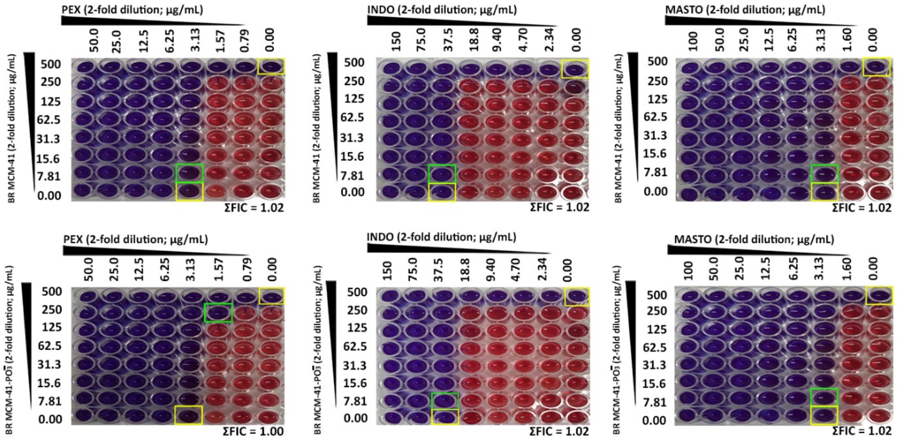

Figure S19. Fractional inhibitory concentration (FIC) index of BR MCM-41 and MCM-41-PO_3^- in combination with antimicrobial peptides (AMPs; PEX, INDO, and MASTO) against *P. aeruginosa* ATCC27853 using checkerboard assay.

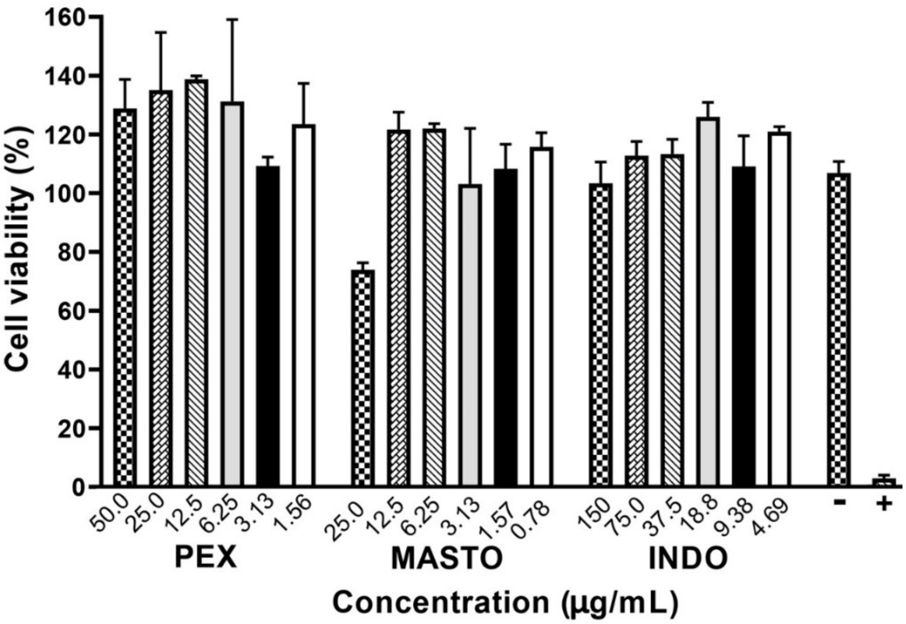

Figure S20. Effects of PEX, INDO, or MASTO on HEK-293 cell viability following 24 h incubation. Statistical analyses were performed using one-way ANOVA followed by Tukey's post-hoc test. No statistically significant differences were found ($p > 0.05$). Data is expressed as mean ± SD ($n = 3$).

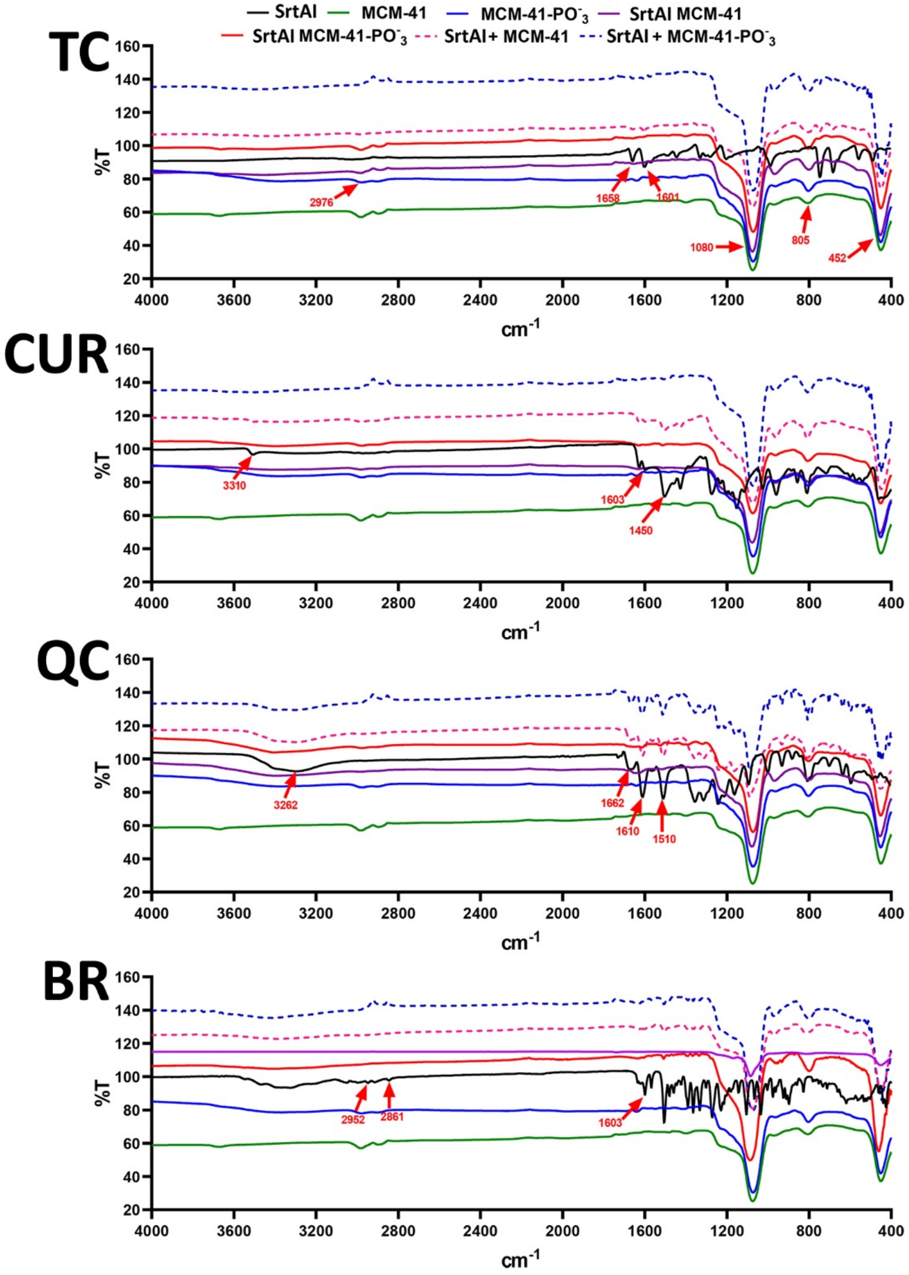

Figure S21. Fourier-transform infrared spectroscopy (FTIR) spectrum of the sortase A inhibitors (SrtAIs, TC, CUR, QC, and BR) alone and loaded into MCM-41 and MCM-41-PO_3^-, and their physical mixture with MCM-41 and MCM-41-PO_3^-.
